# A HLBDA, GA, and COA for optimal operation of distributed energy resources

**DOI:** 10.1371/journal.pone.0340259

**Published:** 2026-01-30

**Authors:** Bilal Naji Alhasnawi, Sabah Mohammed Mlkat Almutoki, Hayder Khenyab Hashim, Abdellatif M. Sadeq, Ali Qasim Almousawi, Basil H. Jasim, Raad Z. Homod, Firas Faeq K. Hussain, Mahmood A. Al-Shareeda, Alžběta Dočekalová, Vladimír Bureš

**Affiliations:** 1 Department of Fuel and Energy Techniques Engineering, Petroleum and Energy Engineering Technical College, Al-Furat Al-Awsat Technical University, Kufa, Iraq; 2 Department of Electricity Techniques, Al-Samawah Technical Institute, Al-Furat Al-Awsat Technical University, Kufa, Iraq; 3 Al-Furat Al-Awsat Technical University, Kufa, Iraq; 4 Independent Researcher, Mechanical Engineering, Doha, Qatar; 5 Faculty of engineering, University of Kufa, Iraq; 6 Electrical Engineering Department, Basrah University, Basrah, Iraq; 7 Department of Oil and Gas Engineering, Basra University of Oil and Gas, Basra, Iraq; 8 College of Engineering, Al-Ayen Iraqi University, Thi-Qar, Iraq; 9 Department of Electronic Technologies, Basra Technical Institute, Southern Technical University, Basra, Iraq; 10 Faculty of Informatics and Management, University of Hradec Králové, Hradec Králové, Czech Republic; Aalto University, FINLAND

## Abstract

Although renewable energy sources offer enormous potential to improve environmental sustainability, maximizing economic benefits inside microgrids requires resolving their intermittency and irregularity. A viable alternative is to combine energy storage with renewable energy technologies. This article introduced a energy management system for hybrid renewable power plants that includes fuel cells, wind turbines, solar cells, battery energy storage devices, and micro-turbines. Optimization problem is formulated as Hyper Learning Binary Dragonfly Algorithm (HLBDA) for optimizing economic benefits and with objectives of minimizing operating costs and pollutant gas emissions. Suggested model is compared with existing methods like Genetic Algorithms (GA), and Crayfish Optimization Algorithm (COA). Also, stochastic framework is considered suitable solution for achieving optimal operation point in microgrids to cope with uncertain parameters. According to the simulation results, suggested method proves reductions in overall system costs and pollutant gas emissions. The proposed system achieved significant superiority across all indicators. In the area of cost reduction, the algorithms demonstrated remarkable progress. The algorithms achieved significant improvements in cost reduction compared to genetic algorithm (GA). HLBDA algorithm achieved a 12.4% cost saving compared to GA, and the COA algorithm showed a 3.24% improvement in cost reduction. In the area of carbon emission reduction, the algorithms also showed significant progress: the HLBDA algorithm recorded the highest emission reduction rate at 9.54%, and the COA algorithm showed a 2.40% improvement in emission reduction.

## 1. Introduction

Despite fact that energy is fundamental engine of human growth, International Energy Agency (IEA) claims that over 1300 million people in rural areas struggle to acquire power from grid due to techno-economic constraints. Conventional power systems that use fossil fuels to produce energy face economic, political, and environmental difficulties due to their significant greenhouse gas emissions [1]. Due to GHG emissions, fossil fuel depletion, and limited grid accessibility in rural areas, utilities are transitioning to Renewable Energy Sources (RESs). Remote locations may be powered by environmentally benign electricity produced by RESs [2].

Environmental concerns have increased interest in Energy Storage Systems (ESSs) and RES worldwide. In order to supply expected demands to end users, microgrids have developed into complex systems that integrate a variety of dispersed generators. Because they address the intermittency and non-dispatchability problems of RESs and increase supply dependability, hybrid systems that integrate RESs with conventional sources are becoming more and more common. Effective energy management systems (EMSs) are necessary to optimize financial and ecological advantages of these hybrid systems [2].

Delivering electricity at the lowest cost while appropriately adapting to supply conditions and load demands is the main goal of an MG EMS. In order to assist efficient short-term planning and lower overall operating costs, EMS may manage energy storage components, perform real-time energy forecasts for RES, and modify loads. In order to lower overall power expenditures, the EMS also seeks to optimize revenue from each MG resource. It guarantees the MG runs profitably and efficiently, improving its long-term profitability and sustainability. Optimization strategies are essential for energy management in MGs, particularly when it comes to maximizing the quantity of energy supply and storage capacity. The complex, multi-objective issues that come up in MG management have dependable solutions thanks to these algorithms. To find best configurations that strike balance between cost, performance, and environmental impact, they efficiently scan vast solution spaces. Because RES are dynamic and demand patterns vary, meta-heuristic algorithms guarantee that energy supplies and storage systems are appropriately sized. Better dependability, lower operating costs, and fewer pollutants are the outcomes of this [3].

This paper’s contribution is suggestion of intelligent planning framework designed to address challenges associated with operating MGs using different RES. The key contribution is developed HLBDA, GA, and COA algorithms to optimize economic benefits and with purposes of minimizing pollutant gas emissions and operating costs. PV, WT, FC, MT, ESS, and smart load participation in energy demand response are all part of the system. It estimates uncertainties such as solar radiation and wind speed using a scenario-based approach.

## 2. Literature review and contributions

**Fig 1:**
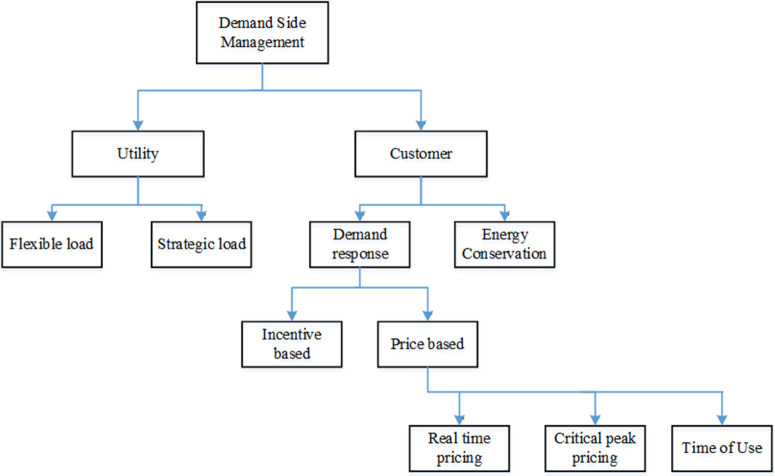
Demand side management classification.

Numerous approaches and goals have been used to handle the microgrid EMS challenge in MG operational planning. This section reviews the relevant papers on day-ahead optimal scheduling of DERs. Different DSM techniques and DRPs were combined in literature to examine how they affected functioning of grid-connected microgrids. Fig 1 depicts a basic classification of DSM programs that are frequently used in research projects [4].

The authors presented optimized microgrid management with intelligent planning based on a chaos theory and salp swarm algorithm for demand response and renewable energy integration in [5]. The authors of [6] presented a workable prototype for an energy management system in a smart microgrid that takes uncertainties into account. The authors of [7] presented clever deep learning approaches for energy management in renewable-based microgrids. The authors of [8] presented a home energy management system that takes into account uncertainty and efficient demand response techniques. Writers of [9] described use of deep reinforcement learning and expert knowledge for EMS in microgrids. Authors in [10] introduced an IoT-based optimization strategy for EMS. The authors of [11] introduced optimization model for scheduling loads in EMS. The authors in [12] discussed how to use EMS in microgrid environment to optimize energy expenses in the residential sector. Writers in [13] proposed the implementation of demand response in distribution networks. The writers presented multi-microgrid optimization-based hierarchical energy management system in [14]. Authors in [15] introduced optimization of residential and industrial DR. Authors presented EMS of the optimal isolated microgrid architecture with reliability and environmental considerations in [16]. The authors provided optimal economic dispatch for micro grids in [17], considering electric, hydrogen, and ESS. The authors introduced the optimization model for EMS in rural households in [18]. In [19], the authors discussed EMS in smart cities to lower peak demand and save energy. The writers in [20] introduced EMS and control technique for green energy-based hybrid system. For a microgrid, the authors of [21] offered the best architecture and energy management while accounting for excess energy and dependability. A sophisticated microgrid energy management method with real-time monitoring interface was introduced via writers of [22]. Writers obtainable effective EMS that takes RES and DR program into account in [23].

Writers in [24] introduced cockroach swarm algorithm technique for apartment EMS. A multi-level optimal EMS for grid-tied microgrid that accounts for demand and weather uncertainty was proposed by the authors of [25]. Using artificial neural network models, the authors of [26] demonstrated efficient EMS in grid-connected microgrids. Authors developed a solid EMS and demand decrease system for loads based on the IoE in [27]. Authors presented home EMS considering RES, ESS, and electric vehicle as backup in [28].

An improved Bald Eagle the authors in [29] introduced a search optimization method for the top energy management systems. Writers of [30] introduced coalition game theory based on consensus algorithms for EMS in microgrids. Writers in [31] introduced a SOA-RBFNN approach for system modeling optimal EMS in grid-connected. In [32], authors introduced EMS for hybrid microgrids established on mixed-integer linear programming. In [33], writers introduced economic dispatch in MG utilizing butterfly optimization technique. Authors’ presentation in [34] was titled scheduling devices with integration of hybrid energy sources utilizing AI algorithms. The authors of [35] discussed how to optimize energy usage for DSM in smart homes using combined operations of microgrids. The authors demonstrated DR for energy-saving homes in [36]. The authors provided flexibility of residential loads for DR provisions in microgrid in [37]. Authors presented distributed energy management of micro grid with high penetration of distributed energy resources based on ADMM in [38].

The authors proposed towards energy efficiency in buildings by utilizing dynamic coordination between households and loads in [39]. EMS consumption in renewable energy fed ecosystems was introduced by the authors of [40]. Artificial neural network-based coordination control for AC/DC smart microgrid was introduced by the authors of [41]. In [42], writers discussed optimization solutions for EMS. The authors presented a control system in [43] for effective scheduling in microgrids with demand response support during emergency situations. The authors in [44] proposed AC/DC microgrid for industries as strategy for effective EMS. Technique for obtaining MPPT for SCADA based on PV systems was provided via writers of [45]. Effective computation for sparse load shifting in EMS was provided by the authors of [46].

Deep reinforcement learning-based EMS of microgrids with uncertainties was introduced by the authors of [47]. In [48], writers offered solution for scheduling problems utilizing bald eagle search optimization technique. Green energy scheduling for smart grid DSM was presented by authors of [49]. Writers in [50] discussed mitigating uncertainty effects on EMS performance for isolated microgrids incorporating tiny producing activities. Adaptive neuro-fuzzy inference approach is used in on-grid/off-grid EMS described by authors of [51]. EMS based on cloud computing and IoT was presented via authors of [52]. The authors introduced Multi-objective DR to RTP in [53] utilizing scheduling approach. When wind, storage, and demand response are present, the authors of [54] employed uncertainty modeling to maximize energy hub performance. Using a cloud computing platform, writers of [55] introduced a brand-smart EMS as service for nanogrid equipment. Writers of [56] introduced real-time optimal scheduling controller for EMS utilizing binary backtracking search technique. The authors of [57] used the grasshopper optimization method to offer the optimal load shedding strategy for islanded power systems with distributed energy supply. A day-ahead market was taken into consideration when the authors of [58] provided resilient optimal microgrid planning based on linear column-and-constraint generation approach.

The authors of [59] introduced ground-breaking real-time electricity scheduling system EMS using IoE. Writers in [60] presented multi-objective scheme for integrated EMS with hybrid sources based optimizing performance. The authors provided an optimization of PV/WT/FC power system architecture for different locations in Libya in [61]. Deep learning-based optimal EMS for integrated residential systems with solar and battery energy storage was introduced by writers of [62]. To reduce carbon emissions, writers of [63] suggested a strategy that makes use of multi-renewable energy systems and artificial intelligence. The authors presented a double-layer microgrid EMS for strategic short-term operation scheduling in [64]. In [65], writers described stochastic energy management and optimal planning of smart microgrid for environmentally friendly transportation and demand-responsive ammonia production. The writers introduced electricity market for decarbonized microgrid in [66].

Authors proposed optimal scheduling for hydrogen-electric coupling systems in [67] while accounting for source-load variability. In [68], the writers discussed EMS of PV/diesel/ESS. The authors in [69] introduced efficient optimization in buildings based on DSM. Writers discussed the impact of PV forecasting on microgrid operation in [70]. ANFIS for reliable and efficient supply chain performance in manufacturing was introduced via the authors of [71].

Writers introduced EMS for microgrid control established on two-level economic model predictive control in [72]. The authors presented a smart EMS communication platform in [73] that uses mixed-integer linear programming. Writers presented hybrid deep learning for EMS for industrial microgrids in [74]. Authors discussed how to optimize demand response in deregulated power markets in [75]. For PV/diesel hybrid systems, authors of [76] proposed a modified energy management strategy based on an artificial intelligence optimizer to reduce fuel consumption. The authors in [77] described effective EMS for households using optimization-based integration of RES in smart grids. In [78], authors minimize energy costs by utilizing an optimization method with hybrid energy storage system. Optimization technique for efficient EMS in a microgrid was introduced by writers of [79]. The authors suggested optimal peak load shifting in buildings utilizing deep clustering reinforcement learning in [80]. The authors in [81] demonstrated coordinated operation of multi-energy sources with consideration for green hydrogen and congestion management using learning technique. The authors in [82] presented artificial neural network-based coordination and control of AC/DC Micro grid. The deep reinforcement learning approach for hierarchical coordination of networked was introduced by the authors of [83]. Raspberry Pi3-based SCADA-controlled home was demonstrated via writers of [84].

### 2.1. Scientific gaps and limitations

Aforementioned literature assessment identified some limitations and gaps in science and understanding.

- In many systems, an EMS for a grid-connected microgrid with various renewable energy resources, including a PV, WT, FC, MT, and BESS not been investigated.
- In many systems, authors did not use an HLBDA to minimize cost and emission reduction.
- In many papers, impact of load forecast uncertainty, possible output of PV, and WT are not computed

### 2.2. The contributions of this study

This artical innovation and originality fill aforementioned scientific gaps.

- This paper suggests EMS for grid-connected microgrid with various renewable energy resources, including PV), WT, FC, MT and BESS.
- In this paper, impact of load forecast uncertainty, possible output of PV, and WT are computed.
- To demonstrate efficacy of the proposed HLBDA in achieving objectives such as energy cost reduction and carbon emission reduction, simulation results are compared with those of GA, and COA.

## 3. Proposed system description

**Fig 2:**
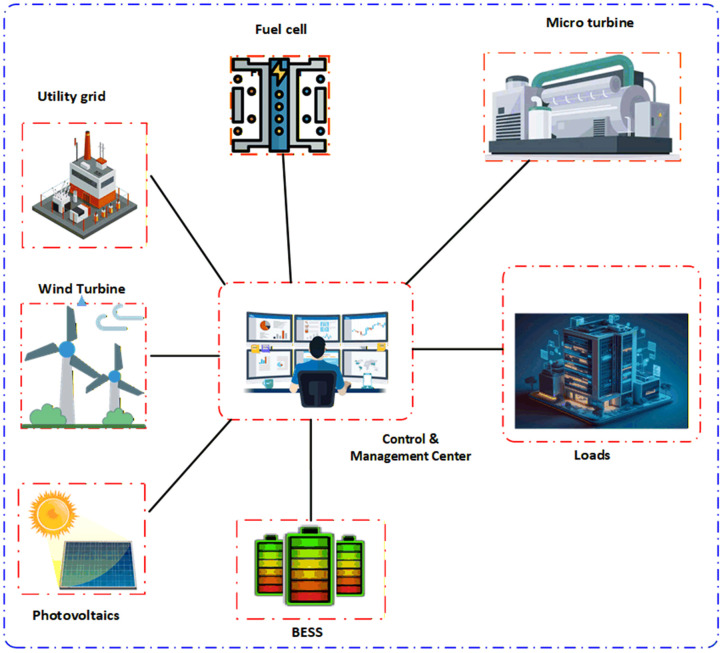
Proposed system.

Fig 2 shows schematic of MG linked to the electrical grid. The MG encompasses variety of DGs, as MTs, FC, WT, PV, and BESS. These sources are all considered to produce active power. Due to decisions made via microgrid operator, power exchange bridge connects grid and the microgrid for intraday electricity trading.

### 3.1. Photovoltaic system

Parameters from photovoltaic (PV) modules are retrieved using a variety of circuit models. The literature describes in detail how a comparable electric circuit can be used to simulate any PV device. Two diodes, two resistances, and a current source that creates current $I_{ph}$ based on incidence of light make up the circuit model employed in this investigation. Whereas Rs represents internal losses brought on by the current flow in the solar cell, ${\text{R}}_{\text{sh}}$ represents the leakage current as a result of ground losses. The two-diode model presented in this study can more accurately capture the nonidealities of a solar panel’s behavior with regard to temperature and the variable nature of irradiance.

In fact, via utilizing Kirchhoff’s current in conjunction with Shockley diode equation of a PV cell’s electrical properties as follows [85]:


$$I_{PV}=I_{ph}-I_{d}-I_{r}$$ (1)

$$I_{d}=I_{d\text{1}}+I_{d\text{2}}=I_{\text{01}}\left(e^{\frac{V_{pV}+R_{R}I_{TV}}{n\text{1}V_{T}}}-\text{1}\right)+I_{\text{02}}\left(e^{\frac{V_{pV}+RI_{TV}}{n\text{2}V_{T}}}-\text{1}\right)$$ (2)

$$I_{r}=\frac{V_{PV}+R_{s}I_{PV}}{R_{sh}}$$ (3)

Given is thermal voltage $V_{T}$:


$$V_{T}=\frac{K\times T}{q}$$ (4)

The operational temperature T and light-related irradiation G are what control how much electric current the solar cells can produce. Consequently, equation (1) becomes:


$$I_{PV}=I_{ph}-\left[I_{\text{01}}\left(e^{\frac{V_{PV}+R_{s}I_{PV}}{n\text{1}V_{T}}}-\text{1}\right)+I_{\text{02}}\left(e^{\frac{V_{PV}+R_{\text{2}}I_{PV}}{n\text{2}V_{T}}}-\text{1}\right)\right]-\frac{V_{PV}+R_{s}I_{PV}}{R_{sh}}$$ (5)

where $I_{ph}$ is the incident light current produced by cell, $I_{\text{01}}$ and $I_{\text{02}}$ represent the diode’s saturation currents, respectively. Temperature of cell is denoted via T, series resistance by $R_{s}$, shunt resistance by $R_{sh}$, the charge of the electron by q, the ideality factor by $n_{\text{1}}$ for diode 1, and ideality factor by $n_{\text{2}}$ for diode 2.

In order to maximize voltage and current output of PV while adhering to the required parameters, series and parallel connections between solar cells are employed. This formula can be used to create and simulate a solar module model in MATLAB/Simulink. Equation (6) describes the PV panel’s output current:


$$\begin{array}{c} I_{PV}=I_{Ph}N_{P}-I_{\text{01}}N_{P}\left\{\text{exp}\left[G_{\text{1}}\left(V_{PV}+N_{S}R_{S}I_{PV}\right)-\text{1}\right]\right\}\\ \;\;\;\;\;\;\;\;\;\;-I_{\text{02}}N_{P}\left\{\text{exp}\left[G_{\text{2}}\left(V_{PV}+N_{S}R_{S}\frac{I_{PV}}{N_{P}}\right)-\text{1}\right]\right\}-\left\{\frac{\frac{V_{PV}}{N_{S}}+N_{S}R_{s}\frac{I_{PV}}{N_{p}}}{R_{sh}\frac{N_{S}}{N_{P}}}\right\} \end{array}$$ (6)

With:, $N_{P}$ is number of cells in parallel, $N_{s}$ is number of cells in series, $G_{\text{1}}=\frac{\text{1}}{n_{\text{1}}V_{T}N_{S}}$ and $G_{\text{2}}=\frac{\text{1}}{n_{\text{2}}V_{T}N_{S}}$.

Following describes photocurrent $I_{ph}$ [86]:


$$I_{ph}=\left\{\frac{G}{G_{STC}}\right\}\left[I_{ph}\;\text{at}\;STC+K_{i}\left(T-T_{STC}\right)\right]$$ (7)

where G and T represent incident temperature and irradiation measured on module, while ${\text{G}}_{\text{STC}}$ and ${\text{T}}_{\text{STC}}$ represent the temperature and irradiation at standard test conditions STC $\left(\frac{\text{1000}\;\text{W}}{{\text{m}}^{\text{2}}},{\text{25}}^{\circ }\text{C}\right)$. An expression for diode saturation current of two diodes, ${\text{I}}_{\text{01}}$ and ${\text{I}}_{\text{02}}$, is


$$I_{\text{01}}=I_{\text{01}\;\text{at}\;STC}{\left(\frac{T}{T_{STC}}\right)}^{\text{3}}\text{exp}\left[\frac{q}{Kn_{\text{1}}}\left(\frac{G_{STC}}{T_{STC}}-\frac{G}{T}\right)\right]$$ (8)

$$I_{\text{02}}=I_{\text{02}}\;\text{at}\;STC{\left(\frac{T}{T_{STC}}\right)}^{\text{3}}\text{exp}\left[\frac{q}{Kn_{\text{1}}}\left(\frac{G_{STC}}{T_{STC}}-\frac{G}{T}\right)\right]$$ (9)

**Fig 3:**
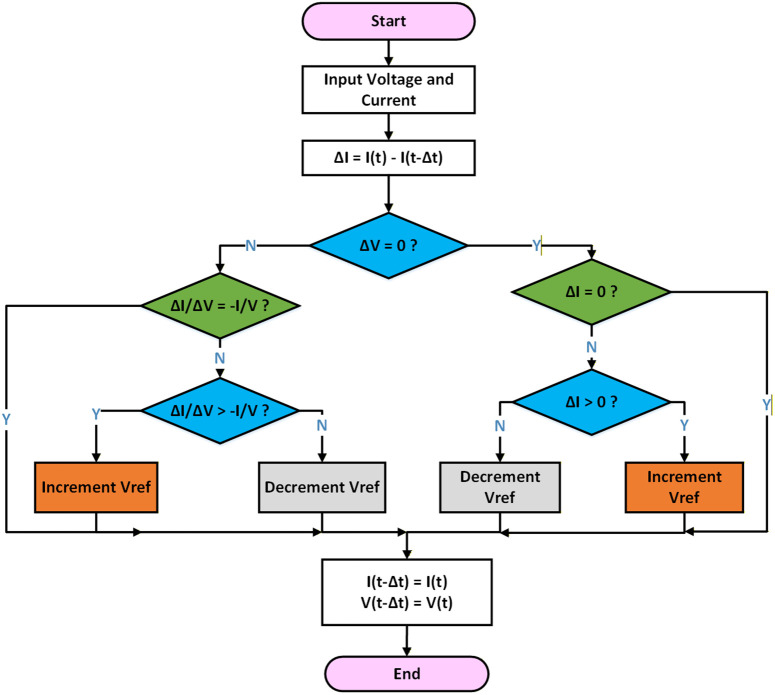
Flowchart for P&O.

An MPPT controller is required to optimize power output and improve PV model performance in the face of weather changes. The standard procedure for managing these controllers is the Perturb and Observe (P&O) software. The PV module’s operating point is disrupted by changing the duty cycle of the PWM signal, which serves as the input for the dc-dc converter. The output power is then measured both before and after the disruption. Duty cycle (D) is the variable control in the P&O algorithm. Its objective is to gradually modify the duty cycle in order to enhance the quantity of power extracted from the PV model. One of the primary tasks of the algorithm is to determine the optimal working posture in order to maximize efficiency. Fig 3 displays the flowchart for the MPPT controller using the conventional P&O technique.

### 3.2. System of wind turbine

The following formulas offer the mathematical model of the suggested wind turbine: An apparatus that converts wind energy, commonly referred to as kinetic energy, to mechanical energy is a WT [87].


$$P_{\text{wind}\;}=\frac{\text{1}}{\text{2}}\rho AC_{p}(\lambda ,\beta )v_{\text{wind}\;}^{\text{3}}$$ (10)

$$\left\{ \begin{array}{ccc} \text{0} & v_{\text{wind}\;}<v_{c} & \;\text{and}\;v_{\text{wind}\;}\geq v_{f}\\ \;\text{PWTmax}\; & & v_{c}<v_{\text{wind}\;}<v_{r}\\ \text{Pr} & & v_{r}<v_{\text{wind}\;}<v_{f}\\ \text{0} & & v_{\text{wind}\;}>v_{f} \end{array}\right.\;$$

where Pr is the rated power output, A, B, and C are constant wind coefficients, $v_{\text{wind}\;}$ is the wind speed, and PWTmax  is the power generated by the wind turbine. The letters $v_{c},v_{r}$, and $v_{f}$ stand for the turbine’s rated, cut-out, and cut-in wind speeds, respectively. By multiplying the speed point proportion by the pitch angle (${\;}^{\circ }\text{C}$), the performance parameter $C_{p}$ is found. This produces the tilt angle, peak motor speed, and rotor blade size (m2). The unit of measurement for air density is kg/m3.

**Fig 4:**
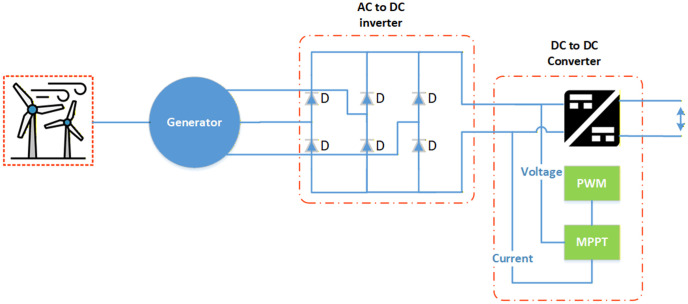
Wind turbine system.

A wind turbine, power converter controlled by MPPT system, and synchronous permanent magnet generator make up wind generator, as shown in Fig 4.

### 3.3. Battery energy storage

Battery has been used as backup energy sources since fluctuating solar radiation and wind speed can affect power output. These battery is used in large-scale ESS due to their affordability, dependability, and user-friendliness. The number of days without interference from power sources is shown by the battery storage, which is modified to meet load requirements. When a battery is under load, this formula arranges its capacity according to autonomous days [88–89]:


$$C_{B}=\frac{\left(D_{L}{\;}^{*}AD\right)}{\left(DOD^{*}\eta_{\text{bat}\;}{\;}^{*}\eta_{\text{inv}\;}\right)}$$ (11)

Here, $C_{B}$ stands for battery capacity, $D_{L}$ for load demand, DOD for depth of discharge, and $\eta_{\text{bat}\;}$ and $\eta_{inv}$ for battery and inverter efficiency.

### 3.4. Modeling of FC

FC claims that chemical energy is transformed to electric energy. Battery and FC processes are identical. However, FC continuously generates DC electricity until it is supplied with hydrogen and oxygen. Fuel cells are used in diesel generator systems because to their ease of use, flexibility, and increased efficiency. Consequently, the FC voltage is computed below [90].


$$v_{FC}=n_{fc}\left[v_{\text{0}}+\frac{U_{g}T}{\text{2}F}\left(\text{ln}\left(\frac{P_{{\text{H}}_{\text{2}}}P_{O_{\text{2}}}^{\frac{\text{1}}{\text{2}}}}{P_{H_{\text{2}}\text{O}}}\right)\right)-r_{i}i_{fc}\right]$$ (12)

where $n_{fc}$ is number of FC in series, $U_{g}$ is universal gas constant, $v_{\text{0}}$ is the voltage of free energy, T is temperature, $i_{fc}$ is current through the FC stack, F is Faraday constant, and $P_{{\text{O}}_{\text{2}}},P_{{\text{H}}_{\text{2}}\text{O}}$ are partial pressures for oxygen, water, and hydrogen, respectively.

### 3.5. Micro turbine modeling

The microturbine’s transfer function model is calculated below:


$$g_{mt}(S)=\frac{\Delta p_{mt}(S)}{\Delta p_{m}(S)}=\frac{k_{mt}}{\text{1}+St_{mt}}$$ (13)

where $t_{mt}$ is micro turbine system’s time constant, $k_{mt}$ is MT system’s gain, $\Delta p_{m}$ is the incremental change in MT power input, and $\Delta p_{mt}$ is the change in MT power output.

## 4. Problem formulation

In characteristic MG, operation management problem is defined as problem of optimal power generation set points and appropriate OFF or ON states to DG units in way that simultaneously minimizes MG’s operating cost and net pollutants emitted within grid while meeting a number of equality and inequality constraints. By way of seen in Fig 2, proposed MG incorporates a number of DG sources, including MT, PV, WT, and a BESS.

Sum of power produced via FC $P_{FC}(t)$, batteries ($P_{Bat}(t)$, solar system ($P_{PV}$), and electrical load is actual power exchanged between hybrid microgrid and utility network $P_{grid}(t)$.


$$P_{grid}(t)=\;P_{FC}(t)+P_{PV}(t)+P_{Bat}(t)+P_{WT}(t)+P_{MT}(t)-P_{Load}(t)$$ (14)

Total operational costs of the MG include fuel prices for DGs, start-up and shut-down costs, and power exchange fees between microgrid and utility. Cost objective function aims to find OPFs from RES to load centers in an inexpensive manner for a given time period. This is how such an objective function is formulated [91]:


$$\begin{array}{c} \alpha_{i}=uW=E,P=W,B=\text{𝒷},\;G=M,m \end{array}$$

$$\begin{array}{cc} \text{Min}{\text{𝒻}}_{\text{1}}(X) & =\;\sum_{t=\text{1}}^{T}\;\;{Price}^{\text{𝓉}}=\sum_{t=\text{1}}^{T}\;\;\{\sum_{\text{𝒾}=\text{1}}^{N_{g}}\;\;[\alpha_{\text{𝒾}}(t)W_{Gi}(t){\text{𝒷}}_{\text{𝒾}}(t)+S_{Gi}\;|\;{\alpha_{\text{𝒾}}}_{\text{𝒾}}(t)\;\;\\ & \;\;-{\alpha_{\text{𝒾}}}_{\text{𝒾}}\left(\text{𝓉}-\text{1}\right)\;|\;]+\sum_{j=\text{1}}^{N_{s}}\;\;[{\alpha_{\text{𝒾}}}_{\text{𝒿}}(t)W_{sj}(t){\text{𝒷}}_{sj}(t)+S_{sj}\;|\;{\alpha_{\text{𝒾}}}_{j}(t)-{\alpha_{\text{𝒾}}}_{\text{𝒿}}\;\\ & \;\;(\text{𝓉}-\text{1})\;|\;]+W_{\text{Grid}\;}(\text{𝓉}){\text{𝒷}}_{\text{Grid}\;}(\text{𝓉})\} \end{array}$$ (15)

where $S_{Gi}$ and $S_{sj}$ are start-up or shut-down costs for ith DG and jth storage, and ${\text{𝒷}}_{G}(t)$ and $B_{Sj}\left(\text{𝓉}\right)$ are bids of DGs and storage devices at hour 𝓉. ${\text{𝒷}}_{\text{Grid}\;}(t)$ is the utility’s bid at time 𝓉, and $W_{\text{Grid}\;}(t)$ is active power that is purchased (sold) from (to) utility at time 𝓉. Active power of units and their connected states are comprised in state variables vector, X, which is definite as:


$$\begin{array}{cc} & X={\left[W_{\text{𝒽}},U_{\text{𝒽}}\right]}_{\text{1}\times \text{2}nT}\\ & W_{\text{𝒽}}=\left[W_{G},W_{S}\right]\\ & \text{𝓃}=N_{\text{𝒽}}+N_{S}+\text{1} \end{array}$$ (16)

where $W_{\text{𝒽}}$ is power vector that includes the active powers of all DGs, $U_{\text{𝒽}}$ is state vector that indicates OFF or ON statuses of all units during each hour of day, n is the number of state variables, and $N_{\text{𝒽}}$ and $N_{\text{𝓈}}$ are total number of generation and storage units. The following is a description of these variables:


$$\begin{array}{cc} & W_{G}=\left[W_{G\text{1}},W_{G\text{2}},\ldots ,W_{G,N_{g}}\right]\\ & W_{Gi}=\left[W_{Gi}(\text{1}),W_{Gi}(\text{2}),\ldots ,W_{Gi}\left(\text{𝓉}\right),\ldots ,W_{Gi}\left(T\right)\right];\text{𝒾}=\text{1},\text{2},\ldots ,N_{\text{𝒽}}+\text{1}\\ & W_{\text{𝓈}}=\left[W_{\text{𝓈}\text{1}},W_{\text{𝓈}\text{2}},\ldots ,W_{\text{𝓈},N_{\text{𝓈}}}\right]\\ & W_{s\text{𝒿}}=\left[W_{sj}(\text{1}),W_{sj}(\text{2}),\ldots ,W_{sj}\left(\text{𝓉}\right),\ldots ,W_{\text{𝓈}j}\left(T\right)\right];\text{𝒿}=\text{1},\text{2},\ldots ,N_{\text{𝓈}} \end{array}$$ (17)

where $P_{Gi}\left(\text{𝓉}\right)$ and $P_{sj}\left(\text{𝓉}\right)$ are actual power outputs of the 𝒾th generator and 𝒿th storage at time  t, and T stands for the total number of hours.


$$\begin{array}{c} \alpha_{g}=\left[\alpha_{\text{1}},\alpha_{\text{2}},\ldots ,\alpha_{n}\right]={\left\{\alpha_{i}\right\}}_{\text{1}\times n}\in \left\{\text{0},\text{1}\right\};\\ \alpha_{k}=\left[\alpha_{k}(\text{1}),\alpha_{k}(\text{2}),\ldots ,\alpha_{k}(t),\ldots ,\alpha_{k}\left(T\right)\right];\;k=\text{1},\text{2},\ldots ,n \end{array}$$ (18)

where $\alpha_{k}(t)$ is status of unit k at hour t.

Environmental footprints caused by air pollution are taken into consideration as the second goal in the following stage. In this sense, the objective function involves three of most significant pollutants: carbon dioxide (${\text{CO}}_{\text{2}}$), nitrogen oxides (${\text{NO}}_{x}$) and sulfur dioxide (${\text{SO}}_{\text{2}}$). Second objective’s mathematical expression is as:


$$\begin{array}{c} \text{Min}{\text{𝒻}}_{\text{2}}\left(X\right)=\;\sum_{\text{𝓉}=\text{1}}^{T}\;\;{\;\text{Emission}\;}^{\text{𝓉}}=\sum_{\text{𝓉}=\text{1}}^{T}\;\;\{\sum_{\text{𝒾}=\text{1}}^{N_{\text{𝒽}}}\;\;\left[\alpha_{\text{𝒾}}\left(\text{𝓉}\right)W_{Gi}\left(\text{𝓉}\right)E_{Gi}\left(\text{𝓉}\right)\right]\;\\ \;\;\;\;\;\;+\sum_{\text{𝒿}=\text{1}}^{N_{s}}\;\;\left[\alpha_{j}(t)W_{sj}\left(\text{𝓉}\right)E_{sj}\left(\text{𝓉}\right)\right]+W_{\text{Grid}\;}\left(\text{𝓉}\right)E_{\text{Grid}\;}\left(\text{𝓉}\right)\} \end{array}$$ (19)

$E_{Gi}(t),E_{sj}\left(\text{𝓉}\right)$, and $E_{\text{Grid}\;}\left(\text{𝓉}\right)$ are defined as quantity of pollutants emitted in $\;\text{kgMWh}{\;}^{-\text{1}}$ for each generator, storage device, and utility at hour t, respectively. Following are these emission variables:


$$E_{G\text{𝒾}}\left(\text{𝓉}\right)={\text{CO}}_{{\text{2}}_{{DG}_{\text{𝒾}}}}+{\text{SO}}_{{\text{2}}_{{DG}_{\text{𝒾}}}}\left(\text{𝓉}\right)+{\text{NO}}_{X_{{DG}_{\text{𝒾}}}}\left(\text{𝓉}\right)$$ (20)

${\text{CO}}_{\text{2}{DG}_{\text{i}}}\left(\text{𝓉}\right),{\text{SO}}_{\text{2}{DG}_{\text{i}}}\left(\text{𝓉}\right)$ and ${\text{NO}}_{x_{{DG}_{\text{i}}}}\left(\text{𝓉}\right)$ represent amounts of ${\text{CO}}_{\text{2}},{\text{SO}}_{\text{2}}$ and ${\text{NO}}_{x}$ emissions from ith DG sources at hour 𝓉.


$$E_{sj}\left(\text{𝓉}\right)={\text{CO}}_{{\text{2}}_{{\text{Storage}\;}_{j}}}\left(\text{𝓉}\right)+{\text{SO}}_{{\text{2}}_{{\text{Storage}\;}_{j}}}\left(\text{𝓉}\right)+{\text{NO}}_{x_{{\text{Storage}\;}_{j}}}\left(\text{𝓉}\right)$$ (21)

The amounts of ${\text{CO}}_{\text{2}},{\text{SO}}_{\text{2}}$ and ${\text{NO}}_{x}$ emissions from jth storage unit during tth hours of day are denoted by ${\text{CO}}_{{\text{2}}_{\text{sorage}\;}}\left(\text{𝓉}\right),{\text{SO}}_{{\text{2}}_{\text{storage}\;}}\left(\text{𝓉}\right)$ and ${\text{NO}}_{x_{\text{storage}\;}}\left(\text{𝓉}\right)$.


$$E_{\text{Grid}\;}(t)={\text{CO}}_{{\text{2}}_{\text{Grid}\;}}\left(\text{𝓉}\right)+{\text{SO}}_{{\text{2}}_{\text{Grid}\;}}\left(\text{𝓉}\right)+{\text{NO}}_{X_{\text{Grid}\;}}\left(\text{𝓉}\right)$$ (22)

The MG’s DGs must generate enough power to meet the grid’s whole demand. There is no immediate need to take into account transmission losses, which are modest numerically, because the paper proposes a tiny 3-feeder radial LV system.


$$\sum_{\text{𝓉}=\text{1}}^{N_{\text{𝒽}}}\;W_{Gi}\left(\text{𝓉}\right)+\sum_{\text{𝒿}=\text{1}}^{N_{\text{𝓈}}}\;W_{sj}\left(\text{𝓉}\right)+W_{\text{Grid}\;}\left(\text{𝓉}\right)=\sum_{k=\text{1}}^{N_{k}}\;W_{Lk}\left(\text{𝓉}\right)$$ (23)

$N_{k}$ is total number of load levels and $W_{Lk}$ is amount of kth load level.

Each DG’s active power output is constrained by following lower and upper constraints for a stable operation:


$$\begin{array}{cc} & W_{Gi,\text{min}}(t)\leq W_{Gi}(t)\leq W_{Gi,\text{max}}\left(\text{𝓉}\right)\\ & W_{sj,\text{min}}(t)\leq W_{sj}(t)\leq W_{sj,\text{max}}\left(\text{𝓉}\right)\\ & W_{\text{grid},\text{min}\;}(t)\leq W_{\text{Grid}\;}(t)\leq W_{\text{grid},\text{max}\;}\left(\text{𝓉}\right) \end{array}$$ (24)

$W_{G,\text{min}}\left(\text{𝓉}\right),W_{s,\text{min}}\left(\text{𝓉}\right)$ and $W_{\text{grid},\text{min}\;}\left(\text{𝓉}\right)$ represent minimal active powers of utility, jth storage, and ith DG at time 𝓉. Similarly, maximum power generation of comparable units at hour 𝓉 is represented by $W_{G,\text{max}}(t),W_{s,\text{max}}(t)$ and $W_{\text{grid},\text{max}\;}(t)$.

Following formula and constraints can be given for a normal battery since there are restrictions on charge and discharge rates of storage devices during each time interval:


$$E_{\text{ess}\;,t}=E_{\text{ess}\;,\text{𝓉}-\text{1}}+\eta_{\text{charge}\;}W_{\text{charge}\;}\Delta \text{𝓉}-\frac{\text{1}}{\eta_{\text{discharge}\;}}W_{\text{discharge}\;}\Delta \text{𝓉}$$ (25)

$$\begin{array}{cc} & \left\{ \begin{array}{c} E_{\text{ess}\;,\text{min}}\leq E_{\text{ess}\;,\text{𝓉}}\leq E_{\text{ess}\;,\text{max}}\\ W_{\text{charge}\;,\text{𝓉}}\leq W_{\text{charge},\;\text{max}};\;W_{\text{discharge},\;\text{𝓉}}\leq W_{\text{discharge},\;\text{max}} \end{array}\right.\; \end{array}$$ (26)

where $W_{\text{charge}\;}\left(W_{\text{discharge}\;}\right)$ is allowed rate of charge (discharge) during specific time period (Δt), $E_{\text{ess}\;,t}$ and $E_{\text{ess}\;,\text{𝓉}-\text{1}}$ are amounts of energy stored inside battery at hour 𝓉 and 𝓉−1, and $\eta_{\text{charge}\;}\left(\eta_{\text{discharge}\;}\right)$ is battery’s efficiency during charge (discharge) process. $W_{\text{charge},\text{max}\;}\left(W_{\text{discharge},\text{max}\;}\right)$ is maximum rate of battery charge (discharge) for each time interval Δ𝓉. $E_{\text{ess},\text{min}\;}$ and $E_{\text{ess},\text{max}\;}$ are lower and upper bounds on quantity of ESS inside battery.

## 5. Modeling uncertainty with scenarios

Stochastic programming is optimization method that offers solution for uncertain problems. The four main approaches that make up this strategy are the approximation analytical method, scenario-based modeling approach, Monte Carlo simulation-based method (MCS), and three-point estimating methodology. The MCS approach’s limitations can be addressed as an approximation using the Taylor series expansion method. The stochastic behaviors of different variables can be described using scenario-based method, which is also used in this study to account for influence of uncertainty [92].

### 5.1. The scenario creation procedure

Each particular feature is given a random number between 0 and 1 in order to generate scenarios. As a result, error that is obtained across associated probability distribution function (PDF) is taken into account when simulating the uncertainties. The solar system’s power outputs, wind power production, load demand, and market price as:


$$P_{WT,t,s}=P_{WT,t}^{\text{forecast}\;}+\Delta P_{WT,t,s};\;WT=\text{1},\ldots ,N_{WT};\;t=\text{1},\ldots ,N_{T};s=\text{1},\ldots ,N_{s}$$ (27)

$$P_{PV,t,s}=P_{PV,t}^{\text{forecast}\;}+\Delta P_{PV,t,s};\;PV=\text{1},\ldots ,N_{PV};\;t=\text{1},\ldots ,N_{T};s=\text{1},\ldots ,N_{s}$$ (28)

$$P_{D.ld,t,s}=P_{D,ld,t}^{\text{forecast}\;}+\Delta P_{d,ld,t};\;ld=\text{1},\ldots ,N_{D};\;t=\text{1},\ldots ,N_{T};S=\text{1},\ldots ,N_{s}$$ (29)

$${\;\text{Price}\;}_{u\;\text{utility}\;,t,s}={\;\text{Price}\;}_{u\;\text{utility}\;,t,s}^{\text{forecast}\;}+\Delta {\;\text{price}\;}_{u\;\text{utility}\;,t,s};\;t=\text{1},\ldots ,N_{T};\;s=\text{1},\ldots ,N_{s}$$ (30)

$P_{WT,t}^{\text{forecast}\;},P_{PV,t}^{\text{forecast}\;}$, $Price\;{\;}_{utility\;,t,s}^{forecast\;},P_{D,ld,t}^{\text{forecast}\;},\Delta P_{WT,t,s},\Delta P_{PV,t,s},\Delta \;price{\;}_{\text{utility}\;,t}$, $s,\Delta P_{d,ld,t},{\;N}_{WT},N_{PV},NDt,N_{s}$ and T are forecasted values of output of WT, forecasted output of PV, ldth forecasted load demand, WT power output forecast error, PV power forecast error of market price forecast, quantities of WT systems, time intervals, quantities of scenarios, and intervals of time.

### 5.2. Scenario reduction procedure

Increasing number of scenarios could improve the model’s accuracy. But this could result in increased consumption. In order to get the best outcomes, scenario-reduction technique is used. For this reason, synchronous backward technique is used. Probability and distance of a scenario pair are represented by $\pi_{s}$ and $DT_{s;s}$, when Ns are considered as distinct scenarios and ξ(s=1,…,Ns). Procedure is as follows:

**Step 1:** Scenarios should be divided into two sets in this process: agreed scenarios (S) and disregarded scenarios (Ds). Algorithm will be executed via decided scenarios. For Ds set, starting value is zero. Following formula is used to determine each scenario pair’s distance.


$$DT_{S,S^{\prime }}=DT\left(\xi_{S},\xi_{S^{\prime }}\right)=\sqrt{\sum_{i=\text{1}}^{d}\;\;\left(x_{i}^{s}-x_{i}^{s^{\prime }}\right)}\;s,s^{\prime }\in S$$ (31)

Step 2: Determine (r,k) scenario pair’s lowest distance using formula below.:


$$DT_{k,r}=\text{min}DT_{k,s^{\prime }}\;k,s^{\prime }\in S;\;s\neq k$$ (32)

Steep 3: Equations (33) and (34) are utilized to calculate $PD_{k,r}$ and $PD_{d}$, as


$$PD_{k,r}=\rho (k)\times DT_{k,r}\;k\in S$$ (33)

$$PD_{d}=\text{min}PD_{k}\;k\in S$$ (34)

Step 4: To create new scenarios, utilize the following equation:


S=S−{d},DS=DS+{d},ρ(r)=ρ(r)+ρ(d) (35)

Step 5: Steps 2, 3, and 4 are continuously repeated until required number of scenarios is reached. Aforementioned backward method can be used to identify ideal scenario set, which can then be taken into consideration for modeling uncertainties of the suggested scheduling problem.

## 6. Artificial intelligence techniques used in EMS

These days, solar and wind power generation are becoming crucial components of microgrids, intelligent buildings, and networks that offer more and more electricity. However, due to high unpredictability, intermittent nature, and unreliability of solar and wind energy, security and dependability of large-scale grid-connected RES have been identified as the most important issues that need to be solved. To ensure grid power stability and power supply security, electrical networks will need to carefully deploy, monitor, and optimize renewable energy sources. AI and other clever tactics are powerful tools that can reduce costs, improve system performance, manage the complexity of the global energy transition, and accelerate the decarbonization process. They are mostly used in EMS, grid operation optimization, renewable energy production, and demand forecasting [93,94].

### 6.1. Genetic algorithm approach

**Fig 5:**
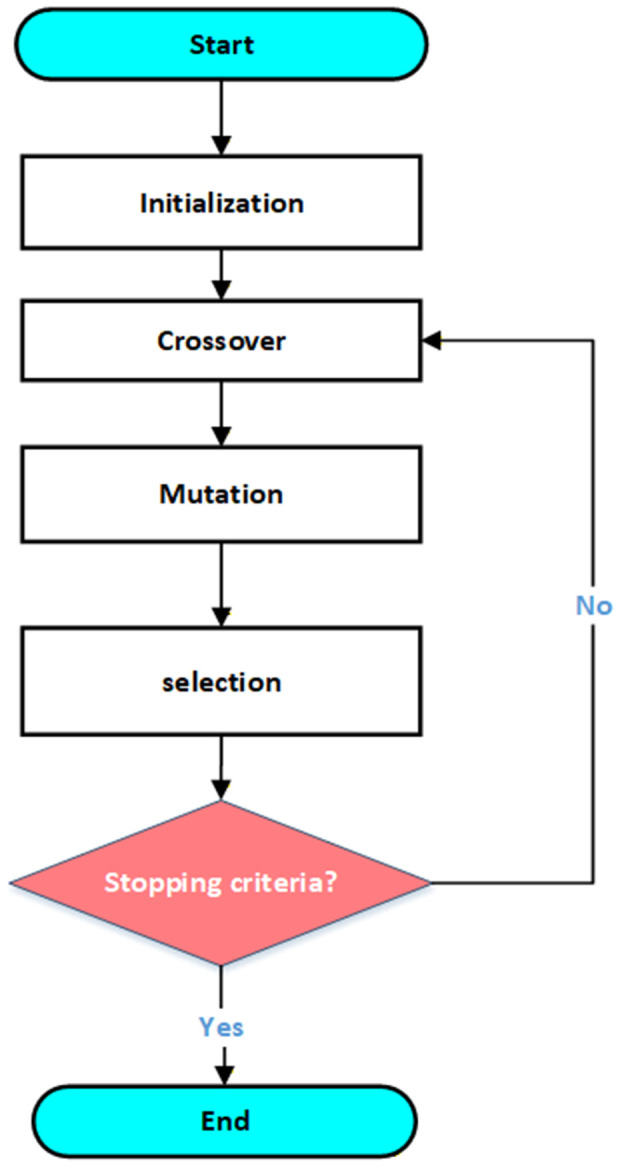
The genetic algorithm’s flowchart.

The four main steps of implementing bill reduction load shifting system using genetic algorithm are as follows: establishing initial population, individual breeding, maintaining genetic diversity, and choosing individuals for next generation (Fig 5) [95].

#### 6.1.1. Initial population.

The technique begins by generating a starting random population that can adhere to all of the restrictions. It starts by filling with people, signifying a potential appliance operation schedule. A random individual who is not a duplicate is substituted for duplicated people in the population. Each person so represents a single potential appliance operation schedule. Each appliance is then given a load at random within the load operation time window. An individual can be thought of as a matrix, with each column representing the periods and each line representing an appliance’s operating schedule.

#### 6.1.2. Crossover.

Crossover process is applied to preceding generation at start of each genetic generation. Initially, individuals from the population pool are randomly crossed in pairs, with each pair consisting of parent 1 and parent 2. Each parent’s response (i.e., the relevant equipment and usage times) is subsequently given to the child. The parents alternate each load that is passed.

An appliance’s operating schedule (pertaining to a certain child) is destroyed if it violates limitations. The most widely used crossover procedures (such cutting points) are ineffective when dealing with matrix persons and complex limitations. Because of this, the current study employed a more deterministic crossover technique in which loads are alternatively transmitted to child between parents from load list sorted based on the loads’ execution times. Crossover strategy that was selected increases the likelihood of having legitimate children.

#### 6.1.3. Mutation.

A local optimum may result from using the crossover strategy. Overcommitting can be achieved by a mutation technique. In this study, the mutation affects a portion of the population that was acquired during the crossover. The proportion of mutation, which is definite as optimization parameter, is used by the algorithm to determine which people will be mutated. When mutation is administered to individual, one randomly chosen load is moved via one period, either to left or right.

#### 6.1.4. Selection.

The algorithm chooses which members of population pool made up of old and new populations—that is, original population of preceding generation and crossing and mutated population, respectively—to pass on to the following generation during the selection phase. Additionally, duplicates are removed from the final unified population. Lastly, each person is assessed using the Fitness Individual (FI), as per equation (36).


$$FI=\frac{\text{1}}{\sum_{p=\text{1}}^{p}\;\;(\left(\left(\sum_{a=\text{1}}^{A}\;\;E_{\text{Demand}(p,a)}-E_{DG}(p)\right)*\text{Eprice}(p)\right)\;}$$ (36)

The appliance’s operation schedule matrix is navigated using both and, for example, cartesian coordinates. If mutated person does not follow all constraints, mutation is reversed and a different load that have not been evaluated for mutation is chosen at random.In general, fitness equation can be thought of as inversion of total energy cost from appliance operation schedule. It is computed via multiplying energy balance via corresponding energy price. Higher fitness ratings therefore indicate lower energy expenditures and are hence better candidates for inheritance.

Following the evaluation of each individual, the algorithm uses the input data to choose n best individuals to pass on to following generation. Moreover, non-elite competitions produce any survivors. In this type of competition, two individuals are selected at random from remaining population based on their fitness ratings. Moreover, non-elite competitions produce any survivors (i.e., population size minus elite size). In this type of competition, two individuals are selected at random from remaining population based on their fitness ratings. Even person with highest fitness score is not always carried down to following generation because likelihood of an individual being chosen increases with fitness score. Additionally, this technique avoids local maximums because the finest individuals are already certain to inherit by elite selection, and a lower fitness individual may create superior individuals.

### 6.2. COA

COA is inspired via crayfish foraging, summer vacations, and competitive behavior. The COA is explored during summer resort stage and exploited during foraging and competition stages. The properties of swarm intelligence optimization are represented by crawfish colony X, which is defined at start of process. Location of i th crayfish, denoted by $X_{i}$, indicates a solution [96].

#### 6.2.1. Initialize population.

In multi-dimensional optimization problem, every crayfish is matrix. Every column matrix signifies the solution to an issue. Every variable $X_{i}$ in set of variables ($X_{i,\text{1}},X_{i,\text{2}}$, $\;\ldots ,X_{i,\text{dim}}\backslash )$ must fall between lower and upper bounds. In order to initialize COA, set of potential solutions X in space are random generated. Population size N and dimension dim serve as foundation for candidate solution X. Equation (37) illustrates how the COA algorithm is initialized.


$$X=\left[X_{\text{1}},X_{\text{2}},\cdots ,X_{N}\right]=[\begin{array}{ccccc} X_{\text{1},\text{1}} & \cdots & X_{\text{1},j} & \cdots & X_{\text{1},\text{dim}}\\ \vdots & \cdots & \vdots & \cdots & \vdots \\ X_{i,\text{1}} & \cdots & X_{i,j} & \cdots & X_{i,\text{dim}}\\ \vdots & \cdots & \vdots & \cdots & \vdots \\ X_{N,\text{1}} & \cdots & X_{N,j} & \cdots & X_{N,\text{dim}} \end{array}]$$ (37)

where dim is population dimension, X is starting population position, N is number of populations, and $X_{i,j}$ represents position of individual i in j dimension. Value of $X_{i,j}$ is derived from Equation (38).


$$X_{i,j}=lb_{j}+\left(ub_{j}-lb_{j}\right)\times \;\text{rand}\;$$ (38)

where $lb_{j}$ stands for jth dimension’s lower bound and $ub_{j}$ for its upper bound.

### 2.6. Foraging stage (exploitation)

If food is too large, crayfish will tear it with its claws before using its second and third walking feet to eat it. Definition of the food location $X_{\text{food}\;}$ is


$$X_{\text{food}\;}=X_{G}$$ (39)

The definition of food size Q is:


$$Q=C_{\text{3}}\times \;\text{rand}\;\times \left(\frac{{\;\text{fitness}\;}_{i}}{{\;\text{fitness}\;}_{\text{food}\;}}\right)$$ (40)

where $C_{\text{3}}$ is food factor, ${\;\text{fitness}\;}_{i}$ signifies fitness value of i th crayfish, and fitness food signifies fitness value of food location.

Largest food item serves as basis for crayfish’s assessment of food size. Food is too large when $Q>\frac{\left(C_{\text{3}}+\text{1}\right)}{\text{2}}$. Crayfish will now use its first claw foot to tear food. Following is the mathematical formula.


$$X_{food}=\text{exp}\left(-\frac{\text{1}}{Q}\right)\times X_{food}$$ (41)

As the meal shreds and shrinks, second and third paws will alternately pick it up and place it in mouth. Additionally, as the food that crayfish collect is likewise correlated with their food consumption, the foraging equation is as follows:


$$X_{i,j}^{t+\text{1}}=X_{i,j}^{t}+X_{\text{food}\;}\times p\times \left(\text{cos}\left(\text{2}\times \pi \times \;\text{rand}\;\right)-\text{sin}\left(\text{2}\times \pi \times \;\text{rand}\;\right)\right)$$ (42)

Crayfish only needs to approach the meal and eat it directly when $Q\leq \frac{\left(C_{\text{3}}+\text{1}\right)}{\text{2}}$.


$$X_{i,j}^{t+\text{1}}=\left(X_{i,j}^{t}-X_{\text{food}\;}\right)\times p+p\times \;\text{rand}\;\times X_{i,j^{*}}^{t}$$ (43)

Depending on size of their food Q, crayfish employ several eating strategies during Foraging stage, with food $X_{\text{food}\;}$ being best option. Crayfish will approach food Q when its size is appropriate for them to consume. Significant difference between optimal and crayish solutions is indicated when Q is too large. As result, $X_{\text{food}\;}$ ought to be decreased and placed closer to food.

### 6.3. Binary Dragonfly Algorithm (BDA)

The BDA, discrete variant of Dragonfly Algorithm (DA), was first introduced via Mirjalili in 2016. As previously stated, this method emulates the natural swarming behavior of dragonflies. Way the dragonflies cooperate to find best solution—finding food supply—and avoid attacker—models exploratory and exploitative mechanisms of DA. A process of updating a location in DA involves five primary behaviors: attraction, cohesiveness, separation, alignment, and distraction [97].

These are descriptions of each of these behaviors:

- Separation, which is act of avoiding a static collision with another person in the neighborhood.
- Alignment, which shows how a person’s velocity matches that of other people in their neighborhood.
- Cohesion, which describes people’s propensity to gravitate toward the neighborhood’s center.

All of a swarm’s members should be attracted to food sources and diverted from any hazards because their main objective is to survive.

The following is the mathematical model for each of these behaviors:

- Separation is determined as [98]:

$$S_{i}=-\sum_{j=\text{1}}^{M}\;X-X_{j}$$ (44)

where M is number of neighboring individuals, $X_{j}$ indicates location of j-th neighboring individual, and X denotes current individual’s position.

- Formula for alignment is as:

$$A_{i}=\frac{\sum_{j=\text{1}}^{M}\;\;V_{j}}{M}$$ (45)

where $X_{j}$ demonstrations velocity of j-th neighbouring individual.

- This formula is used to calculate cohesion:

$$C_{i}=\frac{\sum_{j=\text{1}}^{M}\;\;X_{j}}{M}-X$$ (46)

where M is number of neighborhoods, $X_{j}$ indicates location of j−th neighboring individual, and X  signifies current individual’s position.

Individuals in natural swarms aggressively draw toward food sources and divert attention away from potential predators in addition to separating, aligning, and establishing cohesive groupings.

- The following formula is used to determine attraction to a food source:

$$F_{i}=Xf-X$$ (47)

where Xf is location of food source and X is current individual’s position.

- Following formula is used to calculate an enemy’s distraction:

$$E_{i}=Xe+X$$ (48)

when the enemy’s position is indicated by Xe.

These five behaviors govern the movements of the dragonflies in DA. To update each dragonfly’s position, compute the following step vector:


$$\Delta X_{i}(t+\text{1})=\left(sS_{i}+aA_{i}+cC_{i}+fF_{i}+eE_{i}\right)+w\Delta X_{i}(t+\text{1})$$ (49)

If s denotes the weight of separation, separation of i−th person is indicated by $S_{i}$, cohesion weight is indicated by c, and the alignment weight is represented by a. A cohesion of i−th individual is represented by $C_{i}$, food factor by f, food source by $F_{i}$, enemy factor by e, and iteration timer is t, the inertia weight is w, and enemy location of i−th individual is denoted by $E_{i}$.

A positions of the dragonflies in the original DA are updated using:


$$X_{i}(t+\text{1})=X_{i}(t)+\Delta X_{i}(t+\text{1})$$ (50)

This algorithm’s ability to navigate and move allows it to solve continuous issues.


$$X_{i}^{d}(t+\text{1})=\left\{ \begin{array}{cc} \text{1}-X_{i}^{d}(t) & \;\text{rand}\;<\text{TF}\left(\Delta X_{i}^{d}(t+\text{1})\right)\\ X_{i}^{d}(t) & \;\text{rand}\;\geq TF\left(\Delta X_{i}^{d}(t+\text{1})\right) \end{array}\right.\;$$ (51)

$$\text{TF}(\Delta X)=\left|\frac{\Delta X}{\sqrt{\Delta X^{\text{2}}+\text{1}}}\right|$$ (52)

where $X_{i}^{d}$ is the dth location of the ith dragonfly, TF(ΔX) is the transfer function, and t indicates current iteration. Rand shows an integer that is created at random between 0 and 1. Algorithm 1 displays the BDA pseudocode. During optimization process, the BDA can frequently offer several types of local and global searches with separation, alignment, and cohesion. Due to attraction and distraction, the dragonflies are also able to take advantage of the better possibilities and stay away from the negative ones. Because of these five swarming tendencies, the BDA algorithm performs significantly better.

BDA calculates the varying probability of dragonfly position using V-shaped transfer function. Unlike other binary metaheuristics, BDA does not require dragonfly to select between values of 1 and 0 when using this transfer function. BDA’s high level of exploration has allowed it to identify promising search spaces.

Algorithm 1. Binary Dragonfly Algorithm

$\begin{array}{c} 𝐑𝐚𝐧𝐝𝐨𝐦𝐥𝐲\;𝐢𝐧𝐢𝐭𝐢𝐚𝐥𝐢𝐳𝐞\;𝐩𝐨𝐬𝐢𝐭𝐢𝐨𝐧𝐬\;𝐨𝐟\;𝐍\;𝐝𝐫𝐚𝐠𝐨𝐧𝐟𝐥𝐢𝐞𝐬,\;𝐗\\ 𝐈𝐧𝐢𝐭𝐢𝐚𝐥𝐢𝐳𝐞\;𝐭𝐡𝐞\;𝐬𝐭𝐞𝐩\;𝐯𝐞𝐜𝐭𝐨𝐫𝐬,\;𝐀𝐗\;𝐭𝐨\;𝐳𝐞𝐫𝐨𝐬\\ \begin{array}{c} 𝐰𝐡𝐢𝐥𝐞\;\left(𝐌𝐚𝐱𝐢𝐦𝐮𝐦\;𝐧𝐮𝐦𝐛𝐞𝐫\;𝐨𝐟\;𝐢𝐭𝐞𝐫𝐚𝐭𝐢𝐨𝐧𝐬\;𝐢𝐬\;𝐧𝐨𝐭\;𝐦𝐞𝐭\right)\\ \begin{array}{c} 𝐄𝐯𝐚𝐥𝐮𝐚𝐭𝐞\;𝐟𝐢𝐭𝐧𝐞𝐬𝐬\;𝐯𝐚𝐥𝐮𝐞𝐬\;𝐨𝐟\;𝐝𝐫𝐚𝐠𝐨𝐧𝐟𝐥𝐢𝐞𝐬\\ 𝐔𝐩𝐝𝐚𝐭𝐞\;𝐭𝐡𝐞\;𝐟𝐨𝐨𝐝\;𝐬𝐨𝐮𝐫𝐜𝐞,\;𝐗𝐟\;𝐚𝐧𝐝\;𝐞𝐧𝐞𝐦𝐲,\;𝐗𝐞\\ 𝐔𝐩𝐝𝐚𝐭𝐞\;𝐬,\;𝐚,\;𝐜,\;𝐟,\;𝐞,\;𝐚𝐧𝐝\;𝐰 \end{array}\\ \begin{array}{c} 𝐟𝐨𝐫\;𝐢\;=\;1\;𝐭𝐨\;𝐧𝐮𝐦𝐛𝐞𝐫\;𝐨𝐟\;𝐝𝐫𝐚𝐠𝐨𝐧𝐟𝐥𝐢𝐞𝐬,\;𝐍\\ 𝐂𝐚𝐥𝐜𝐮𝐥𝐚𝐭𝐞\;𝐒,\;𝐀,\;𝐚𝐧𝐝\;𝐂\\ \begin{array}{c} 𝐂𝐨𝐦𝐩𝐮𝐭𝐞\;𝐅\;𝐚𝐧𝐝\;𝐄\\ 𝐔𝐩𝐝𝐚𝐭𝐞\;𝐬𝐭𝐞𝐩\;𝐯𝐞𝐜𝐭𝐨𝐫\\ \begin{array}{c} 𝐔𝐩𝐝𝐚𝐭𝐞\;𝐩𝐨𝐬𝐢𝐭𝐢𝐨𝐧\;𝐨𝐟\;𝐝𝐫𝐚𝐠𝐨𝐧𝐟𝐥𝐲\;\left(𝐢-𝐭𝐡\right)\\ 𝐞𝐧𝐝\;𝐟𝐨𝐫\\ \begin{array}{c} 𝐞𝐧𝐝\;𝐰𝐡𝐢𝐥𝐞\\ 𝐎𝐮𝐭𝐩𝐮𝐭:\;𝐁𝐞𝐬𝐭\;𝐟𝐨𝐨𝐝\;𝐬𝐨𝐮𝐫𝐜𝐞,\;𝐗𝐟 \end{array} \end{array} \end{array} \end{array} \end{array} \end{array}$

#### 6.3.2. *HLBDA.*

This section applies HLBDA. HLBDA employs hyperlearning technique that integrates the concepts of personal best and personal worst solutions to update location. The dragonflies use the classic BDA to coordinate global worst solution and global best solution for attraction and diversion. Addition person’s best and worst dragonflies to these drills is said to increase the chances of finding food and avoiding enemies.

The following equations are used to determine the attraction and distraction of HLBDA, in contrast to BDA.


$$F_{i}=\frac{\left(Xpb_{i}-X_{i}\right)+\left(Xf-X_{i}\right)}{\text{2}}$$ (53)

$$E_{i}=\frac{\left(Xpw_{i}+X_{i}\right)+\left(Xe+X_{i}\right)}{\text{2}}$$ (54)

If Xpw displays the individual’s worst dragonfly, Xpb represents dragonfly’s position, Xf denotes food supply, and Xe denotes adversary.

Furthermore, the hyperlearning technique allows dragonflies to learn from best replies in the world as well as from their own throughout the search phase.. Instead of changing its position to accommodate swarming behaviors, dragonfly attempts to imitate the most significant historical events as well as its own.

Position of dragonfly is updated in proposed HLBDA in the following way:


$$X_{i}^{d}(t+\text{1})=\left\{ \begin{array}{cc} {\bar{X}}_{i}^{d} & \text{0}\leq r_{\text{1}}<pl\\ Xpb_{i}^{d}(t) & pl\leq r_{\text{1}}<gl\\ Xf^{d}(t) & gl\leq r_{\text{1}}\leq \text{1} \end{array}\right.\;$$ (55)

$${\bar{X}}_{i}^{d}=\left\{ \begin{array}{cc} \text{1}-X_{i}^{d}(t) & r_{\text{2}}<TF\left(\Delta X_{i}^{d}(t+\text{1})\right)\\ X_{i}^{d}(t) & r_{\text{2}}\geq TF\left(\Delta X_{i}^{d}(t+\text{1})\right) \end{array}\right.\;$$ (56)

tindicates current iteration, d stands for number of choice variables (dimension), Xf represents food supply, I represents the dragonfly’s order, and Xpb represents the location of the individual’s best dragonfly. The constant numbers in [0,1] represent personal learning rate (pl) and the global learning rate (gl).

Equation (55) indicates that pl and gl were crucial to learning process. The algorithm will largely look for global and personal best solutions if pl and gl are too low, which makes it prone to being stuck in local optima. On other hand, if pl and gl values are excessively high, position update procedure will probably resemble BDA. Therefore, choices made for pl and gl are quite crucial. HLBDA pseudocode is shown in Algorithm 2.

Algorithm 2. Hyper Learning Binary Dragonfly Algorithm

$\begin{array}{c} 𝐑𝐚𝐧𝐝𝐨𝐦𝐥𝐲\;𝐢𝐧𝐢𝐭𝐢𝐚𝐥𝐢𝐳𝐞\;𝐩𝐨𝐬𝐢𝐭𝐢𝐨𝐧𝐬\;𝐨𝐟\;𝐍\;𝐝𝐫𝐚𝐠𝐨𝐧𝐟𝐥𝐢𝐞𝐬,\;𝐗\;\\ 𝐈𝐧𝐢𝐭𝐢𝐚𝐥𝐢𝐳𝐞\;𝐭𝐡𝐞\;𝐬𝐭𝐞𝐩\;𝐯𝐞𝐜𝐭𝐨𝐫𝐬,\;𝐀𝐗\;𝐭𝐨\;𝐳𝐞𝐫𝐨𝐬\\ \begin{array}{c} 𝐰𝐡𝐢𝐥𝐞\;\left(𝐌𝐚𝐱𝐢𝐦𝐮𝐦\;𝐧𝐮𝐦𝐛𝐞𝐫\;𝐨𝐟\;𝐢𝐭𝐞𝐫𝐚𝐭𝐢𝐨𝐧𝐬\;𝐢𝐬\;𝐧𝐨𝐭\;𝐦𝐞𝐭\right)\\ \begin{array}{c} 𝐟𝐨𝐫\;𝐢\;=\;1\;𝐭𝐨\;𝐧𝐮𝐦𝐛𝐞𝐫\;𝐨𝐟\;𝐝𝐫𝐚𝐠𝐨𝐧𝐟𝐥𝐢𝐞𝐬,\;𝐍\\ 𝐂𝐚𝐥𝐜𝐮𝐥𝐚𝐭𝐞\;𝐭𝐡𝐞\;𝐟𝐢𝐭𝐧𝐞𝐬𝐬\;𝐯𝐚𝐥𝐮𝐞\;𝐝𝐫𝐚𝐠𝐨𝐧𝐟𝐥𝐲\;\left(𝐢-𝐭𝐡\right)\\ 𝐔𝐩𝐝𝐚𝐭𝐞\;𝐩𝐞𝐫𝐬𝐨𝐧𝐚𝐥\;𝐛𝐞𝐬𝐭\;𝐝𝐫𝐚𝐠𝐨𝐧𝐟𝐥𝐲,\;𝐗𝐩𝐛𝐢\; \end{array}\\ \begin{array}{c} 𝐔𝐩𝐝𝐚𝐭𝐞\;𝐩𝐞𝐫𝐬𝐨𝐧𝐚𝐥\;𝐰𝐨𝐫𝐬𝐭\;𝐝𝐫𝐚𝐠𝐨𝐧𝐟𝐥𝐲,\;𝐗𝐩𝐰𝐢\\ 𝐞𝐧𝐝\;𝐟𝐨𝐫\\ \begin{array}{c} 𝐔𝐩𝐝𝐚𝐭𝐞\;𝐟𝐨𝐨𝐝\;𝐬𝐨𝐮𝐫𝐜𝐞,\;𝐗𝐟\;𝐚𝐧𝐝\;𝐞𝐧𝐞𝐦𝐲,\;𝐗𝐞\\ 𝐔𝐩𝐝𝐚𝐭𝐞𝐬,\;𝐚,\;𝐜,\;𝐟,\;𝐞,\;𝐚𝐧𝐝\;𝐰\\ \begin{array}{c} 𝐟𝐨𝐫\;𝐢\;=\;1\;𝐭𝐨\;𝐧𝐮𝐦𝐛𝐞𝐫\;𝐨𝐟\;𝐝𝐫𝐚𝐠𝐨𝐧𝐟𝐥𝐢𝐞𝐬,\;𝐍\\ 𝐂𝐚𝐥𝐜𝐮𝐥𝐚𝐭𝐞\;𝐒,\;𝐀,\;𝐚𝐧𝐝\;𝐂\\ \begin{array}{c} 𝐂𝐨𝐦𝐩𝐮𝐭𝐞\;𝐅\;𝐚𝐧𝐝\;𝐄\\ 𝐔𝐩𝐝𝐚𝐭𝐞\;𝐬𝐭𝐞𝐩\;𝐯𝐞𝐜𝐭𝐨𝐫\\ \begin{array}{c} 𝐔𝐩𝐝𝐚𝐭𝐞\;𝐩𝐨𝐬𝐢𝐭𝐢𝐨𝐧\;𝐨𝐟\;𝐭𝐡𝐞\;𝐝𝐫𝐚𝐠𝐨𝐧𝐟𝐥𝐲\;\left(𝐢-𝐭𝐡\right)\\ 𝐞𝐧𝐝\;𝐟𝐨𝐫\\ \begin{array}{c} 𝐞𝐧𝐝\;𝐰𝐡𝐢𝐥𝐞\\ 𝐎𝐮𝐭𝐩𝐮𝐭:\;𝐁𝐞𝐬𝐭\;𝐟𝐨𝐨𝐝\;𝐬𝐨𝐮𝐫𝐜𝐞,\;𝐗𝐟 \end{array} \end{array} \end{array} \end{array} \end{array} \end{array} \end{array} \end{array}$

Following subsections provide detailed discussion of main steps of the suggested HLBDA:

6.3.2.1. *Initialization:* As can be seen here, the HLBDA randomly initializes population of dragonflies after setting the settings:


$$X=[\begin{array}{cccc} X_{\text{1}}^{\text{1}} & X_{\text{1}}^{\text{2}} & \cdots & X_{\text{1}}^{D}\\ X_{\text{2}}^{\text{1}} & X_{\text{2}}^{\text{2}} & \cdots & X_{\text{2}}^{D}\\ \vdots & \vdots & \ddots & \vdots \\ X_{N}^{\text{1}} & X_{N}^{\text{2}} & \cdots & X_{N}^{D} \end{array}]\to [\begin{array}{cccc} \text{0} & \text{1} & \cdots & \text{0}\\ \text{1} & \text{0} & \cdots & \text{1}\\ \vdots & \vdots & \ddots & \vdots \\ \text{0} & \text{0} & \cdots & \text{1} \end{array}]$$ (57)

Table 1 demonstrations HLBDA’s parameter settings.

**Table 1:** HLBDA parameter configuration.

Parameter	Value
Global rate, gl	0.7
Personal rate, pl	0.4
Number of dimensions, D	Each dataset’s feature count
Number of dragonflies, N	10
Maximum iterations, T	100

6.3.2.2. Fitness evaluation: Every dragonfly is assessed iteratively using a predetermined objective function. Quality of solutions is evaluated using the objective function. This article’s objective function is selected to decrease number of specified features while maximizing classification accuracy. Here is how the objective function looks like:


$$↓\;\text{Fit}\;=\alpha ER+\beta \left(\frac{\left|S\right|}{\left|O\right|}\right)$$ (58)

ER is classification error, |O| is length of original features, and |S| is length of sub-set of selected features. To show/prioritize effects of feature size and classification error on the objective function, two weight infectors are available: α∈[0,1] and β=(1−α).

## 7. Simulations results

This part assesses performance of proposed model on standard test system. In this section, proposed EMS simulation results are showed. The program’s chief objects are to reduce electricity costs, and pollutant gas emissions via shortening wait times. This article suggests that 24-h schedule offers good middle ground between these goals. Outcomes of the Hyper Learning Binary Dragonfly Algorithm (HLBDA), Genetic Algorithms (GA), and Crayfish Optimization Algorithm (COA) are compared in order to verify accuracy of system.

This study successfully developed integrated model for managing microgrid energy under conditions of uncertainty, with the results showing significant variation in the performance of the five algorithms used. The HLBDA algorithm came out on top with exceptional performance, followed by the GA algorithm, then COA.

The Hyper Learning Binary Dragonfly Algorithm achieved a remarkable advantage across all indicators, recording the lowest operating cost, resulting in a 12.4% saving compared to the traditional GA algorithm. This superiority was not accidental, but rather resulted from algorithm’s exceptional ability to achieve optimal balance between different generation sources.

On the environmental side, HLBDA demonstrated a significant commitment to reducing emissions, recording a 9.54% reduction in carbon emissions compared to the GA algorithm. This achievement reflects the algorithm’s efficiency in managing high-emission energy sources and reducing reliance on them.

Regarding the efficiency of renewable energy utilization, the HLBDA algorithm achieved a renewable energy utilization rate of 27.34% of the total energy generated. This percentage is considered excellent given the weather fluctuations and instability of generation from renewable sources. Interestingly, the algorithm was able to achieve this level of utilization while maintaining grid stability, adapting to sudden changes in solar and wind power production without affecting the quality of supply. In field of battery energy storage management, HLBDA recorded a utilization rate of 77.21% of the stored and discharged energy. This high efficiency resulted from optimizing scheduling of battery charging and discharging in accordance with market electricity prices. The algorithm successfully identified the optimal times to charge batteries when electricity prices were low and discharge them during peak hours when prices were high, significantly contributing to reduced operating costs.

The Crayfish Optimization Algorithm ranked third in terms of cost and emissions. This algorithm was distinguished by its ability to effectively utilize renewable energy sources, achieving 25.67% renewable energy utilization. The battery utilization efficiency in the Crayfish Optimization Algorithm was 69.14%. These results reflect acceptable performance, but it falls short of the stronger competitors, as the algorithm faced some difficulties in achieving the optimal balance between different generation sources.

The GA algorithm came in last place in terms of cost and carbon emissions. The renewable energy percentage was 24.81%, with a battery efficiency of 67.82%. This modest performance reflects the limitations of traditional algorithms in dealing with complex multi-objective and constrained problems.

During peak hours from 5 to 8 PM, HLBDA demonstrated superior load management, relying more heavily on batteries and reducing dependence on the main grid. This intelligent strategy resulted in a 14.1% cost saving during peak hours compared to the genetic algorithm. In the area of energy exchange with grid, HLBDA was able to reduce amount of energy purchased from grid while increasing amount sold. This efficient performance contributed to lowering the net energy consumption from grid.

**Fig 6:**
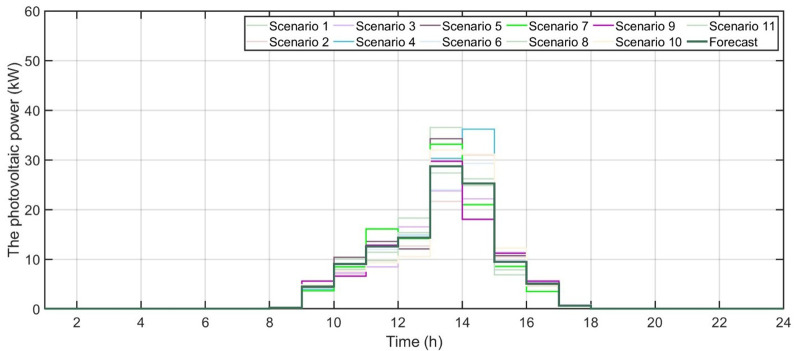
Forecasted PV power output.

**Fig 7:**
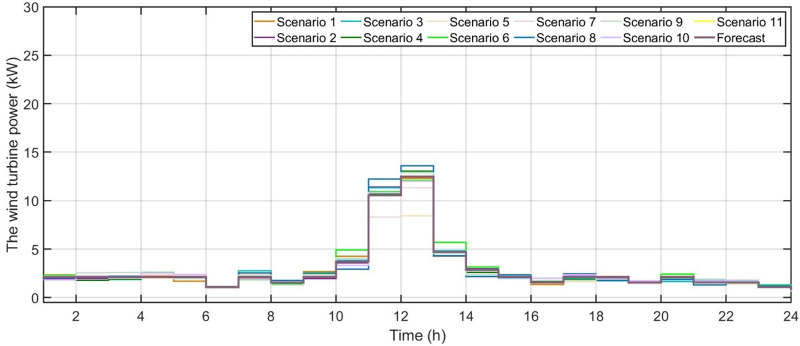
Forecasted WT power output.

**Fig 8:**
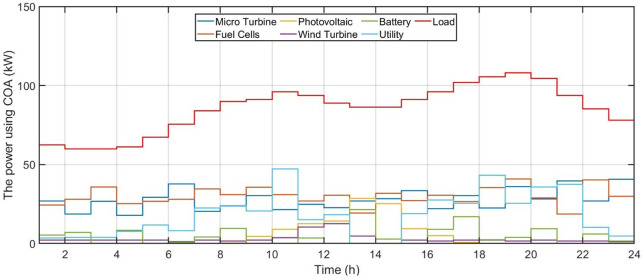
The power of microturbine, photovoltaics, battery, load, fuel cells, wind turbine and utility using Crayfish Optimization Algorithm.

**Fig 9:**
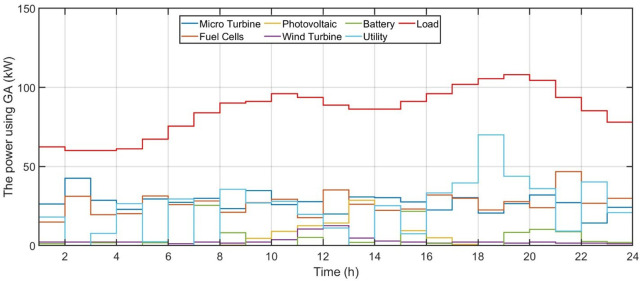
The power of microturbine, photovoltaics, battery, load, fuel cells, wind turbine and utility using GA.

**Fig 10:**
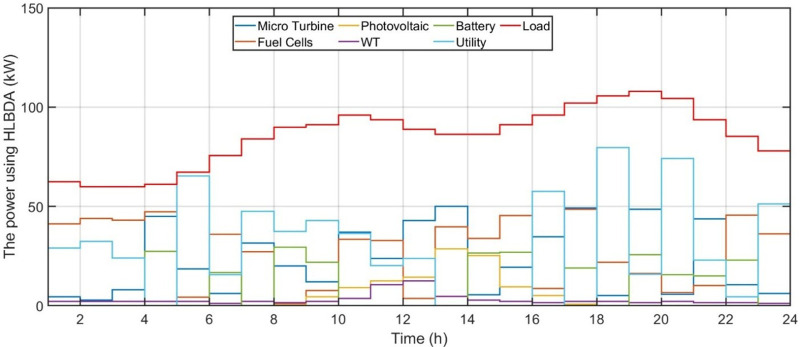
The power of microturbine, photovoltaics, battery, load, fuel cells, wind turbine and utility using HLBDA.

**Fig 11:**
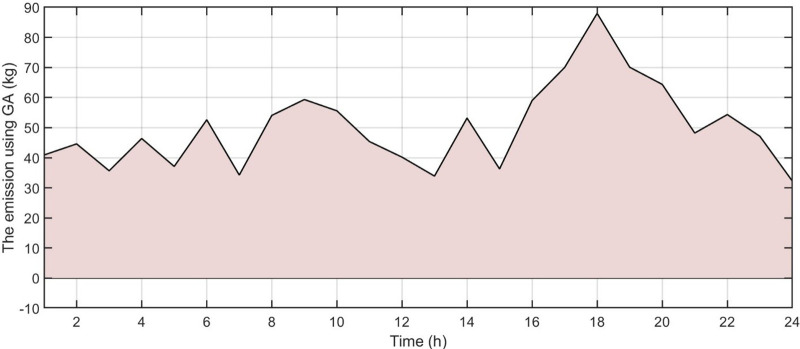
The emmission using GA.

**Fig 12:**
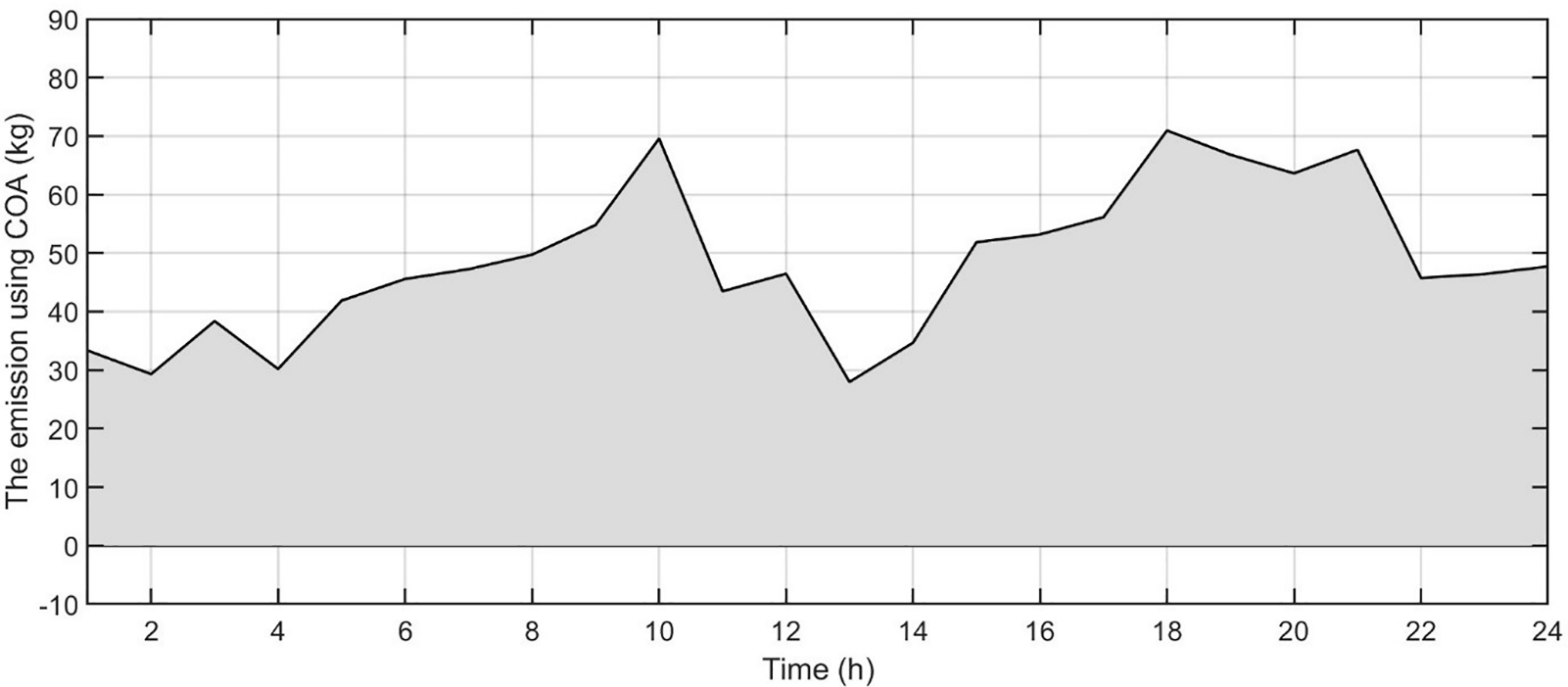
The emmission using COA.

**Fig 13:**
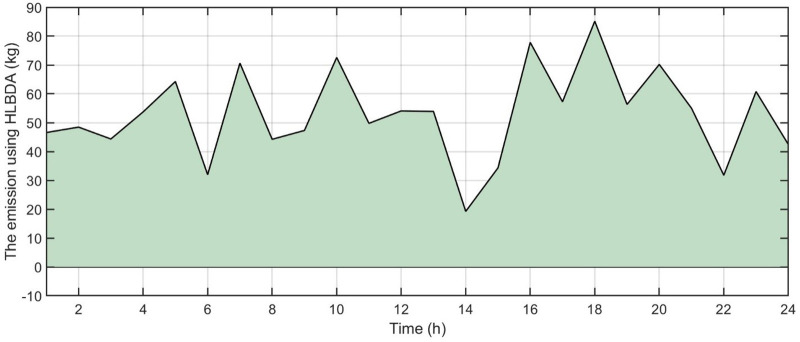
The emmission using HLBDA.

**Fig 14:**
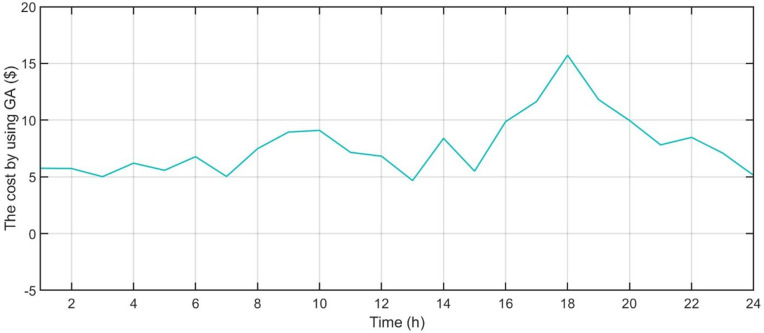
The cost by using genetic algorithm.

**Fig 15:**
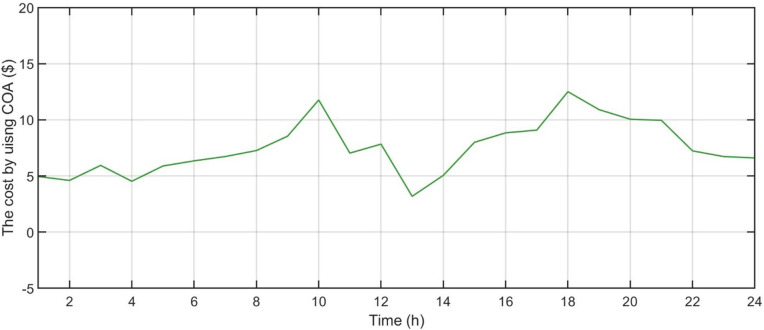
The cost by using crayfish optimization algorithm.

**Fig 16:**
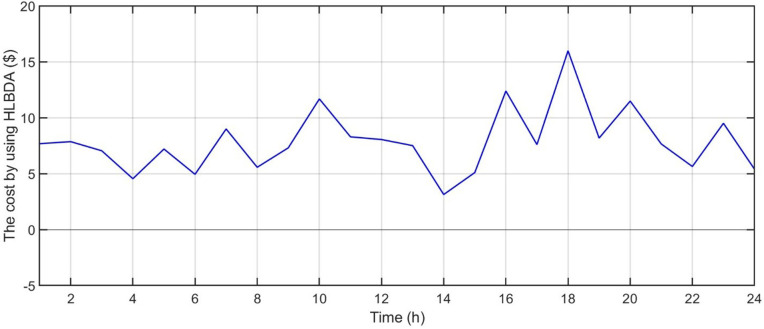
The cost by using HLBDA.

Fig 6 shows the forecasted PV power output. Fig 7 shows the forecasted WT power output. Fig 8 shows the power of microturbine, photovoltaics, battery, load, fuel cells, wind turbine and utility using COA. Fig 9 shows the power of microturbine, photovoltaics, battery, load, fuel cells, wind turbine and utility using GA. Fig 10 shows the power of microturbine, photovoltaics, battery, load, fuel cells, wind turbine and utility using HLBDA. Fig 11 shows the emission using GA. Fig 12 shows the emission using COA. Fig 13 shows the emission using HLBDA. Fig 14 shows the cost of using the genetic algorithm. Fig 15 shows cost of using crayfish optimization algorithm. Fig 16 shows cost of using HLBDA.

## 8. Conclusion

EMS for hybrid renewable power plants that includes DG, ESS, and controlled loads has been presented in this study. This EMS’s three main goals are to assure compliance with system operating limitations, decrease overall operating cost associated with various system components, and lower costs and pollutant gas emissions arising from utility grid operation. Additionally, the stochastic framework is seen to be a good way to deal with uncertain parameters in microgrids by reaching the ideal operating point. Simulation experiments have successfully proven the application of suggested EMS inside microgrid configuration that includes ESS, a PV source, fuel cells, micro-turbines, and aggregate controlled loads.

GA and COA are used to compare the optimization results that were obtained. The suggested demand-side optimization challenge results in lower operating costs and carbon emissions. Regarding efficiency of renewable energy utilization, the HLBDA algorithm achieved a renewable energy utilization rate of 27.34%, with a battery utilization rate of 77.21% of the stored and discharged energy. Whereas COA achieved a renewable energy utilization rate of 25.67%, with a battery utilization rate of 69.14%. The GA algorithm ranked last in terms of cost and carbon emissions. Its renewable energy utilization rate was 24.81%, with a battery efficiency of 67.82%.

We propose using P2P energy trading market model based on time-driven prospect theory to improve study in future work.

## Acknowledgments

The financial support of the project “Application of dynamic system models for ensuring power substation systems cyber security”, n. TS01020105, granted by the Technology Agency of the Czech Republic within the Theta programme, is gratefully acknowledged.
